# Why Are There So Few *Rickettsia conorii conorii*-Infected *Rhipicephalus sanguineus* Ticks in the Wild?

**DOI:** 10.1371/journal.pntd.0001697

**Published:** 2012-06-19

**Authors:** Cristina Socolovschi, Jean Gaudart, Idir Bitam, Thi Phong Huynh, Didier Raoult, Philippe Parola

**Affiliations:** 1 Faculté de Médecine, Université de la Méditerranée, Unité de Recherche en Maladies Infectieuses et Tropicales Emergentes (URMITE), UMR CNRS-IRD 6236, WHO Collaborative Center for Rickettsial Diseases and Other Arthropod-Borne Bacterial Diseases, Marseille, France; 2 Biostatistic Research Unit, LERTIM, EA 3283 Aix-Marseille University, Faculty of Medicine, Marseille, France; 3 Service d'Ecologie et des Systèmes Vectoriels, Institut Pasteur d'Algérie, Algiers, Algeria; University of Texas Medical Branch, United States of America

## Abstract

**Background:**

*Rickettsia conorii conorii* is the etiological agent of Mediterranean spotted fever, which is transmitted by the brown dog tick, *Rhipicephalus sanguineus*. The relationship between the *Rickettsia* and its tick vector are still poorly understood one century after the first description of this disease.

**Methodology/Principal Findings:**

An entomological survey was organized in Algeria to collect ticks from the houses of patients with spotted fever signs. Colonies of *R. conorii conorii*-infected and non-infected ticks were established under laboratory conditions. Gimenez staining and electron microscopy on the ovaries of infected ticks indicated heavy rickettsial infection. The transovarial transmission of *R. conorii conorii* in naturally infected *Rh. sanguineus* ticks was 100% at eleven generations, and the filial infection rate was up to 99% according to molecular analyses. No differences in life cycle duration were observed between infected and non-infected ticks held at 25°C, but the average weight of engorged females and eggs was significantly lower in infected ticks than in non-infected ticks. The eggs, larvae and unfed nymphs of infected and non-infected ticks could not tolerate low (4°C) or high (37°C) temperatures or long starvation periods. *R. conorii conorii*-infected engorged nymphs that were exposed to a low or high temperature for one month experienced higher mortality when they were transferred to 25°C than non-infected ticks after similar exposure. High mortality was observed in infected adults that were maintained for one month at a low or high temperature after tick-feeding on rabbits.

**Conclusion/Significance:**

These preliminary results suggest that infected quiescent ticks may not survive the winter and may help explain the low prevalence of infected *Rh. sanguineus* in nature. Further investigations on the influence of extrinsic factors on diapaused *R. conorii*-infected and non-infected ticks are required.

## Author Summary

The bacterium *Rickettsia conorii conorii* is the etiological agent of Mediterranean spotted fever (MSF), which is a life-threatening infectious disease that is transmitted by *Rhipicephalus sanguineus*, the brown dog tick. *Rh. sanguineus*-*R. conorii conorii* relationships in the wild are still poorly understood one century after the discovery of the disease. In this study, we collected naturally infected ticks from the houses of people afflicted by MSF in Algeria. Colonies of both infected and non-infected ticks were maintained in our laboratory, and we studied the effect of temperature variations on the infected and non-infected ticks. We did not observe any major differences between the biological life cycle of the infected and non-infected ticks held at 25°C. However, a comparatively higher mortality relative to the control group was noticed when *R. conorii conorii*-infected engorged nymphs and adults were exposed to a low temperature (4°C) or high temperature (37°C) for one month and transferred to 25°C. *R. conorii conorii*-infected *Rh. sanguineus* may maintain and serve as reservoirs for the *Rickettsia* if they are not exposed to cold temperatures. New populations of ticks might become infected with *Rickettsiae* when feeding on a bacteremic animal reservoir.

## Introduction

**Figure 1:**
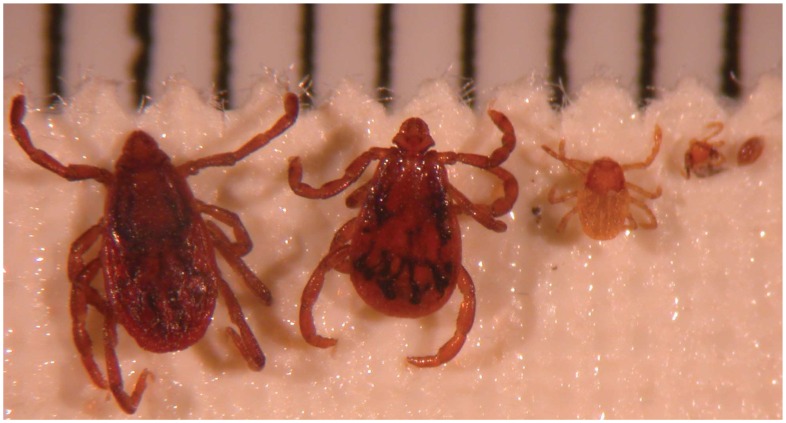
*Rhipicephalus sanguineus*, the vector and potential reservoir of Mediterranean spotted fever caused by *Rickettsia conorii*. A mm scale is at the top of this image.

*Rickettsia conorii conorii* is the etiological agent of Mediterranean spotted fever (MSF), one of the oldest recognized vector-borne infectious diseases [1]. In the 1930s, the brown dog tick, *Rhipicephalus sanguineus*, was suspected to be the vector of MSF. Ticks were crushed and used to inoculate humans who consequently contracted MSF [1], [2]. Crushed eggs, larvae, nymphs, unfed adults collected in the winter and adults obtained from infected *Rh. sanguineus* females were able to infect humans. These data suggested that transstadial transmission (transfer of bacteria from stage to stage) but also transovarial transmission (TOT, the transfer of bacteria from adult female ticks to the subsequent generation of ticks via the eggs) of *R. conorii conorii* occurs in ticks and consequently that *Rh. sanguineus* (Figure 1) could act not only as a vector but also as a reservoir of *R. conorii conorii* [1].

*Rh. sanguineus* has become one of the most globally widespread ticks because of its specialized feeding and association with domestic dogs [3]. Although *Rh. sanguineus* rarely feeds on humans, particularly in temperate countries, it seems to have a greater human affinity in warmer temperatures [4]. This tick is highly adapted to warm climates but also thrives in dog kennels and human homes. It can be imported with dogs to the cooler regions and survive in peridomestic environments, provided that it encounters acceptable conditions. Thus, *Rh. sanguineus* has spread globally between 50°N and 35°S [3]. However, MSF due to *R. conorii conorii* is known to be endemic in North Africa and southern Europe. MSF has also been described in a few countries in sub-Saharan Africa, and a few cases have also been sporadically reported in northern and central Europe [5], [6], sometimes followed by the installation of a local focus of the disease [1]. In contrast, *R. conorii conorii* infection has never been described in the Americas [7].

Although *Rh. sanguineus*-*R. conorii conorii* relationships were a focus of interest of pioneering rickettsiologists, they are still poorly understood even one century later. Interestingly, it has been suggested that *R. conorii conorii* has a negative effect on the survival of its tick vector when *Rh. sanguineus* ticks are experimentally infected [8]–[12]. However, preliminary data have recently demonstrated that naturally infected colonies of *Rh. sanguineus* can be maintained in laboratory conditions over several generations [13]. Therefore, a significant population of infected ticks should exist in the wild. However, the prevalence in the wild of ticks infected by *R. conorii* is low (usually less than 1%). For example, none of the 2,229 *Rh. sanguineus* ticks collected from Spain were positive for *R. conorii* [14]. Rarely, a high prevalence of infected ticks has been found in small foci. For example, when a spotted fever focus was investigated in France in May 2007, 18% (24/133) of the *Rh. sanguineus* ticks collected from the walls of one house and from a garden were found to be infected with *R. conorii conorii* [4].

The vectorial capacity of ticks depends on several characteristics of tick biology, including longevity, host-seeking behavior and mobility, all of which are influenced by extrinsic factors, including climatic conditions [15]. Temperature is known to influence tick-microorganism relationships and consequently, the vectorial capacity of ticks. For example, the maintenance and multiplication of parasites (e.g., *Theileria* spp. and *Babesia* spp.) in ticks has been shown to be influenced mostly by temperature [15]. Moreover, it has been shown that ambient temperatures in excess of 27°C are not permissive for the transmission of *Borrelia burgdorferi*, the agent of Lyme disease in *Ixodes dammini* ticks [16].

In 1972, Injeyan et al. [17] inoculated guinea pigs with infected crushed *Rh. sanguineus* nymphs that were previously held at different temperatures. These ticks were experimentally infected with the so-called “*R. conorii* Simko isolate” isolated from *Rh. simus* collected from cattle in Ethiopia. The clinical reactions were most evident in the guinea pigs injected with nymphs held at 35°C, and the reactions were milder in those held at 5°C, 15°C, 20°C, or 25°C [17]. However, the relevant literature offers epidemiological analyses of the influence of climatic factors on tick-borne rickettsial diseases, rather that laboratory results [4], [17], [18]. For example, during the 1970s, an increase in the number of observed MSF cases was correlated with a decrease in the number of frost days during the preceding year in France [19]–[21].

The aim of this study was to assess some of the life cycle parameters of infected and non-infected *Rh. sanguineus*, the transstadial and transovarial transmission of *R. conorii conorii* and the influence of high and low temperature on the survival of *Rh. sanguineus* infected with *R. conorii conorii*.

## Materials and Methods

We studied the transmission of *R. conorii conorii* in *Rh. sanguineus* through more than twelve generations by using molecular tools and following the life cycle of infected and non-infected ticks under laboratory conditions. Our investigation of the influence of high and low temperature on the survival of several stages of *Rh. sanguineus* infected with *R. conorii conorii* mimicked the cold weather (4°C) and hot summer (37°C) in our area and compared the experimental temperatures to laboratory conditions (25°C).

### *Rhipicephalus sanguineus* infected with *Rickettsia conorii conorii*

**Figure 2:**
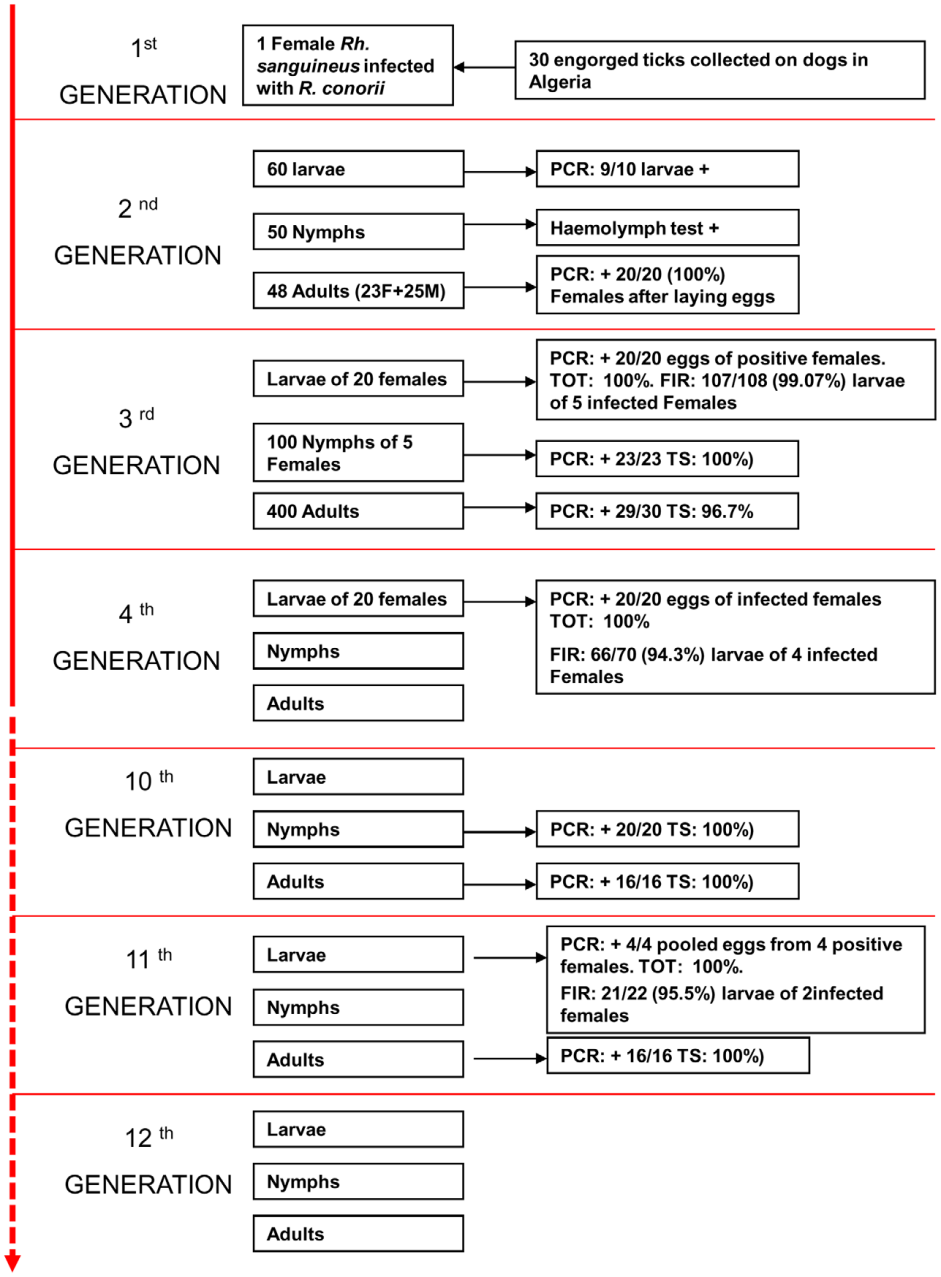
Study of the vertical transmission of *Rickettsia conorii conorii* in naturally infected *Rhipicephalus sanguineus* ticks. PCR, polymerase chain reaction. TS, transstadial transmission (transfer of bacteria from stage to stage). TOT, transovarial transmission, the proportion of infected females giving rise to at least one positive egg or larva. FIR, the filial infection rate, the proportion of infected eggs or larvae obtained from an infected female. M: male. F: female.

**Figure 3:**
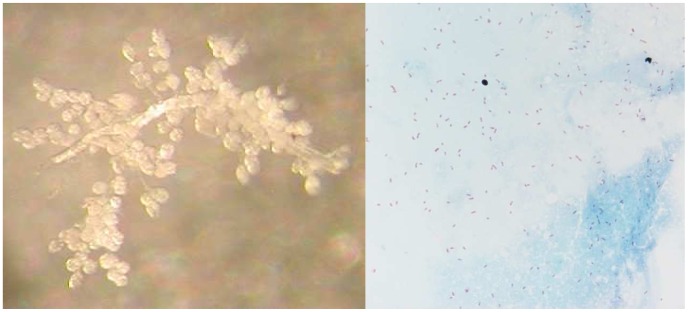
*Rhipicephalus sanguineus* salivary glands and detection of *Rickettsia conorii conorii*. Salivary glands of a Rhipicephalus sanguineus infected with *Rickettsia conorii conorii* (left). Gimenez staining: smears of infected *Rhipicephalus sanguineus salivary* glands (right).

**Figure 4:**
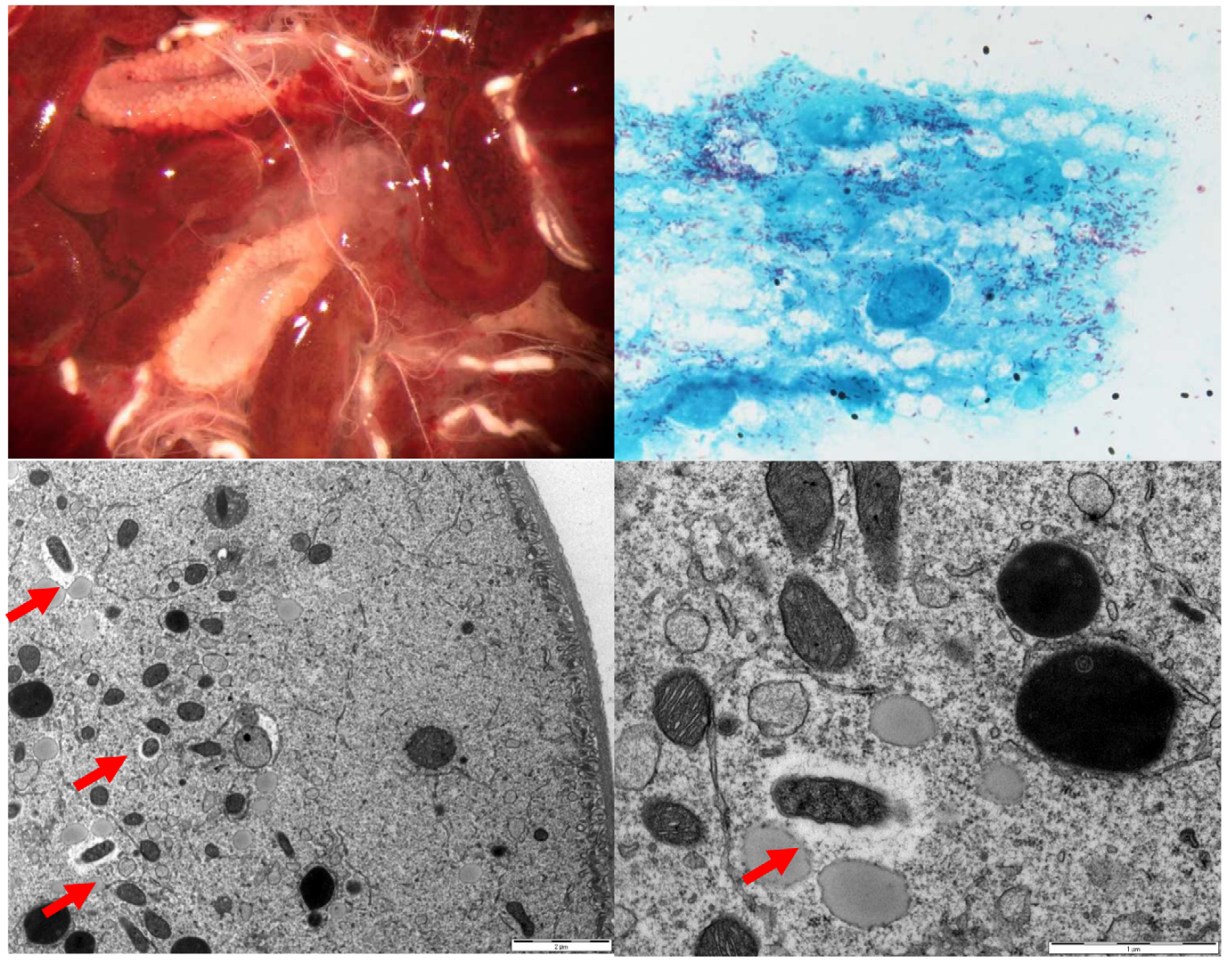
*Rhipicephalus sanguineus* ovary and detection of *Rickettsia conorii conorii*. Ovaries of a *Rickettsia conorii conorii*-infected engorged *Rhipicephalus sanguine*; scale bar: 2 µm (top left). Gimenez staining, smears of infected *Rhipicephalus sanguineus* ovaries (top right). An electron photomicrograph of ovarian tissue from a *Rickettsia conorii conorii*-infected engorged female *Rhipicephalus sanguineus* (bottom left and right). Red flash, *Rickettsia conorii conorii* surrounded by electron-lucent halos. Scale bar: 0.1 µm.

**Figure 5:**
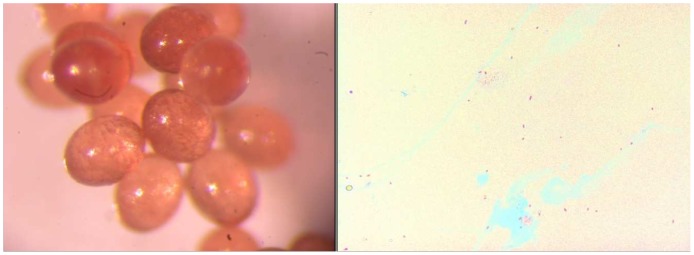
*Rhipicephalus sanguineus* eggs and detection of *Rickettsia conorii conorii*. The eggs of a *Rhipicephalus sanguineus* infected with *Rickettsia conorii conorii*(left). Gimenez staining, the crushed eggs of a *Rhipicephalus sanguineus* infected with *Rickettsia conorii conorii* (right).

To collect *Rh. sanguineus* ticks naturally infected by *R. conorii conorii*, an entomological survey was organized in Algeria. The houses of patients who had contracted MSF between July and August of 2006 were visited. The owners were interviewed about the presence of ticks on their dogs and in their house. When available, engorged females were removed from dogs. The ticks were transported to Marseille, France and stored in environmental incubators at 25°C and 80% relative humidity (RH) with a day/night photoperiod of 16∶8 (L∶D) h [9]. After the ticks laid eggs, DNA was extracted from each tick, and all samples were tested by PCR for the rickettsial *glt*A and *romp*A genes, as previously described [9]. For all PCR procedures, the negative controls consisted of distilled water or DNA extracted from non-infected ticks from laboratory colonies that were added to the PCR master mix. The amplified products were sequenced, analyzed by BLAST (www.ncbi.nlm.nih.gov/blast/Blast.cgi), and compared to those in the GenBank database. A single specimen tested positive for both rickettsial genes, and the analyzed sequences indicated *R. conorii conorii* fragments (result section). The larvae and all subsequent stages of the infected tick were placed on New Zealand white rabbits (*Oryctolagus cuniculus*) that were used as the host for the blood meal [13]. Ticks were placed in each of two cloth ear bags, which were secured with Elastoplast® to the ears of rabbit [9]. Unfed adults from the 2^nd^ generation were used for definitive morphological identification by a researcher (PP) using standard taxonomic keys for adult ticks [22]. To confirm species identification, amplification of the mitochondrial 12S rRNA gene was achieved by conventional PCR [23]. Specimens (larvae, nymphs and adults) of the 3^rd^, 4^th^ and 10^th^ subsequent generations (Figure 2) were tested by real-time (RT)-PCR in a Lightcycler (Roche) instrument for the presence of *Rickettsia* spp. DNA using primers and Taqman probes targeting a partial sequence of the citrate synthase *glt*A gene, as previously described [24]. Gimenez staining, as previously described, was used to highlight morphological structures compatible with *R. conorii conorii* in the salivary glands (Figure 3A), ovaries (Figure 4A) and eggs (Figure 5A) of infected *Rh. sanguineus* [25].

### Electron microscopy of ovaries from *R. conorii conorii*-infected *Rh. sanguineus*

Engorged, infected ticks were dissected under a binocular microscope. The ovaries were washed with PBS and fixed overnight in 2% glutaraldehyde in a 0.1 M cacodylate buffer. After being washed in a 0.1 M cacodylate buffer, the specimens were post-fixed in 1% osmium tetroxide in 0.1 M potassium ferricyanide for 1 h and dehydrated in an ascending series of ethanol concentrations ranging from 30% to 100%. After the absolute ethanol dehydration step, the dehydration was finished in propylene oxide. The samples were embedded in Epon 812 resin. Sections (70-nm thick) were stained with 5% uranyl acetate and lead citrate before examination using a transmission electron microscope (Philips Morgagni 268D). For better visualization of the carbohydrate layer, another series was completed using ruthenium red.

### *Rickettsia*-free *Rh. sanguineus* colonies

We used colonies of ticks free of *Rickettsia*, *Ehrlichia*, *Anaplasma*, *Bartonella* and *Coxiella burnetii* originating from Algeria that were morphologically and molecularly [23] identified as *Rh. sanguineus* and had been maintained in our laboratory in an incubator at 25°C with 80% relative humidity since 2006 [26]. When obtained, the 12S RNA mitochondrial sequence data presented 100% similarity to *Rh. sanguineus* from the USA (HM014443), Portugal (FJ536554), and Switzerland (AF483241). Individual New Zealand white rabbits (*Oryctolagus cuniculus*) were used for the attachment of non-infected ticks, as described above. Periodically, new ticks from the wild that tested negative by PCR were included in our non-infected *Rh. sanguineus* colony, as previously described [27].

### Study of the biological parameters of the life cycle (duration of their metamorphosis) of *R. conorii conorii*-infected *Rh. sanguineus*

The life cycle or developmental period of non-infected and *R. conorii conorii*-infected *Rh. sanguineus* ticks was studied through several generations. The duration of the larval, nymphal, and adult feeding (the number of days from placement of the rabbit until drop-off) were studied. In addition, the molting period covering the transition from larvae to nymphs and from nymphs to adults (the number of days from drop-off to ecdysis), the pre-oviposition period (the period from female drop-off to the beginning of oviposition), and incubation periods (from the beginning of oviposition until hatching of larvae), as previously described [27], were studied. The sum of the days of all of these parameters represents the total life cycle of *R. conorii conorii*-infected ticks. The weight of engorged females and the eggs of these females of non-infected and *R. conorii conorii*-infected *Rh. sanguineus* ticks was measured with an analytical balance (XB 620M, Micromega groupSoframe). The weight data of engorged females and eggs were analyzed with GraphPad Prism™ v 2.0 software (La Jolla, USA, www.graphpad.com/prism/Prism.htm).

### Influence of temperature on *R. conorii conorii*-infected *Rh. sanguineus*

Batches of randomly selected eggs, larval and nymphal stage unfed or engorged ticks (N = 100) and adult stage ticks (N = 40), either infected or non-infected, were used for each of three experiments from the 8^th^, 9^th^, and 10^th^ generations. Each of the treatment groups of engorged ticks were held at a particular temperature (4°, 25°, 37°C) for one month and then all of the ticks were held at the same temperature (25°C) for an additional month. The non-engorged ticks held at 4°C, 25°C and 37°C for one month were attached to New Zealand white rabbits for feeding. The experiment with infected and non-infected ticks had been conducted in the same time. The relative humidity (80% RH) was the same for all groups with a light/dark photoperiod of 16∶8 h. The following biological parameters were recorded after one month for infected and non-infected engorged nymphs held at 25°C: the number that were dead without molting, the number that had molted but were dead, and the total number of dead nymphs. For adult ticks, the following biological parameters were noted: the number of ticks dead before attachment on the rabbit, the number dead after attachment, and the total number of dead ticks. Each experiment was performed in triplicate. The infected and non-infected ticks of the corresponding temperature groups were compared. The numbers of dead ticks of each group were compared using a χ2 test conducted with Epi Info software, version 3.4.1 (CDC, Atlanta, USA). Statistical significance was defined as p<0.05.

### Ethics

The animals were handled according to the rules of French Decree N.8 87–848 of October 19, 1987, Paris. Each colony of non-infected and infected ticks had individual rabbits. For the non-infected ticks of the laboratory tick colony, a rabbit was used a maximum for three times for feeding. However, for the infected laboratory colonies and for the analysis of temperature on *R. conorii conorii*-infected and non-infected *Rh. sanguineus* (experimental analysis), an individual rabbit for each batch and for each temperature condition was used only once. All experimental protocols were reviewed and approved by the Institutional Animal Care and Use Committee of the Université de la Méditerranée (Marseille, France).

## Results

### Transmission of *R. conorii conorii*-over twelve generations of *Rh. sanguineus*

A total of thirty engorged female *Rh. sanguineus* ticks were collected from 7 dogs of patients who contracted MSF in Algeria. A single specimen collected in Ghazonet, Algeria tested positive for *R. conorii conorii* (GenBank, accession number *omp*A: DQ518245 and *glt*A: AE008677). The positive controls tested positive for all PCR reactions, and no signal was obtained from the negative controls for any PCR reaction. Molecular identification of tick species based on partial 12S rRNA mitochondrial sequence data indicated 99.7% (337/338) similarity to *Rh. sanguineus* from the USA (HM014443), Portugal (FJ536554), and Switzerland (AF483241).

Twelve successive generations were obtained, and the infected colony is still growing in our laboratory as of this writing (Figure 2). The PCR assay was positive for specimens of all stages of these generations for rickettsial detection. For the 10^th^ generation, all engorged nymphs (20/20), all adults randomly chosen (16/16), 4/4 females after laying eggs and the pools of eggs from these females tested positive by RT-PCR for rickettsial DNA. These data suggest 100% transstadial and transovarial transmission of *R. conorii conorii* in *Rh. sanguineus* ticks. The filial transmission rate (FIR, proportion of infected eggs or larvae obtained from an infected female) of *R. conorii conorii* was 99.07% (107/108), 94.3% (66/70) and 95.5% (21/22) in larvae from several infected females of the 3^rd^, 4^th^, and 11^th^ generations, respectively. Gimenez staining revealed many morphological structures compatible with *R. conorii conorii* in the salivary glands (Figure 3B), ovaries (Figure 4B) and eggs (Figure 5B). Electron microscopy of the ovarian tissue revealed heavy infection compatible with *R. conorii conorii* (Figure 4C, 4D). The *Rickettsiae* exhibited typical rickettsial morphology and size, as previously described [10].

### Study of biological parameters of the life cycle (duration of their metamorphosis) of *R. conorii conorii*-infected *Rh. sanguineus*

**Figure 6:**
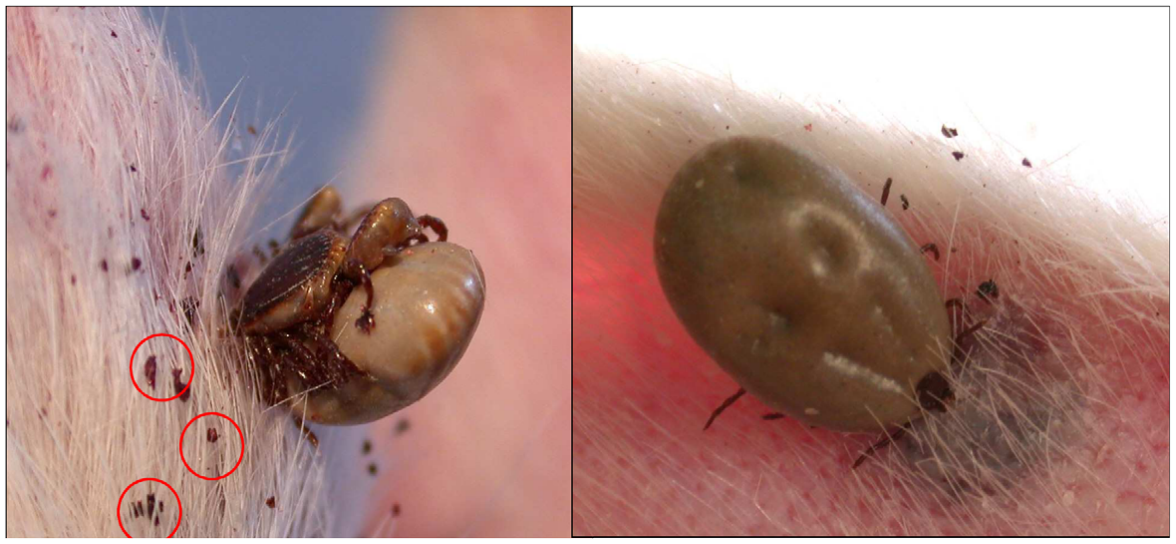
*Rhipicephalus sanguineus* ticks during blood feeding on rabbits. *Rhipicephalus sanguineus* infected with *Rickettsia conorii conorii* during blood feeding on a New Zealand white rabbit, feces circled (left). Inflammation around a bite site of a female *Rhipicephalus sanguineus* infected with *R. conorii conorii* during feeding (right).

No difference was observed between the duration of life cycle of *R. conorii conorii*-infected and non-infected ticks held at 25°C, but the average weight of engorged females and eggs was found to be significantly lower in infected ticks. The number of infected females began to drop off on day 8 after placement on the rabbit, and their blood meal was completed by day 15 (Figure 6). The average weight of 31 infected engorged females was 0.38111 g (range, 0.2518–0.5389 g) compared to 0.4749 g (range, 0.2333–0.6203 g) for six non-infected engorged females (p = 0.0136). At 25°C, the pre-oviposition period started between one and two weeks after the end of female engorgement. The average weight of eggs from one female infected tick was 0.2099 g (range, 0.0998–0.2976 g, 31 samples) compared to 0.2919 g (range, 0.1321–0.4271 g, 6 samples) for non-infected eggs (p = 0.0021). The infected eggs began hatching within 2–3 weeks, as did non-infected eggs. Hatched larvae were kept at 25°C for at least 2–3 weeks before feeding. The duration of pre-feeding period was 2 to 4 weeks after the end of eclosion of the last specimen. Infected larvae fed for 3–6 days. The infected engorged larvae molted into the nymphal stage between 9 and 15 days after engorgement. Molted *R. conorii conorii*-infected nymphs were fed for approximately 3–4 weeks after ecdysis, as were non-infected nymphs. Nymphs were fed for 4–7 days. It took 2–3 weeks for engorged nymphs to molt into adults. Under our standard laboratory conditions, the life cycle of infected *Rh. sanguineus* ticks lasted 18 to 24 weeks. To avoid different abnormalities in infected ticks and to maintain genetic diversity, new, non-infected male ticks were placed on rabbits during the feeding of infected female ticks. In conclusion, under laboratory conditions we did not find any difference in the duration of developmental stages of the life cycle of *R. conorii conorii*-infected *Rh. sanguineus* when compared to non-infected ticks [27].

### Influence of temperature on *R. conorii conorii*-infected *Rh. sanguineus*

#### *Eggs, larvae and unfed nymphs*

The same experiment was performed with several batches of eggs, larvae and unfed nymphs of infected and non-infected ticks. None of these stages, either infected or non-infected, could tolerate low (4°C) or high (37°C) temperatures for one month (data not shown). Moreover, at 25°C the larvae and unfed nymphs did not survive more than one and two months, respectively. To avoid high mortality, larvae and nymphs were placed on the rabbit to feed after 2–3 weeks and 3–4 weeks, respectively.

#### *Engorged nymphs*

In the 3 experiments with engorged nymphs, 88–100% of the *R. conorii conorii*-infected nymphs that had been previously maintained at 4°C died after being transferred to 25°C (53–80% died without molting) compared to 41% (62/150) of non-infected nymphs after one month (288/300 vs. 62/150, p = 0.0001). In contrast, there was no mortality rate difference for the ticks, both infected and non-infected, that had been maintained at 25°C (2.3–2.6%, 8/300 vs. 7/300, respectively). Significantly more engorged, infected nymphs maintained at 37°C died than non-infected nymphs maintained at the same temperature (17–67%, 101/300 and 4–7%, 15/300, respectively, p = 0.0001) (Table 1).

**Table 1:** The effect of temperature on *R. conorii conorii*-infected and non-infected engorged *Rhipicephalus sanguine* nymphs.

Engorged NYMPHS							
	Non-infected			*R. conorii*-infected			p values (total deaths non-infected/infected)
	N. of dead nymphs	N. of dead adults	Total deaths (%)	N. of dead nymphs	N. of dead adults	Total deaths (%)	
	**1 month at 4°C then 25°C for 1 month**						
Exp.1	32/100	0	32/100	71/100	29/100	100/100	
Exp.2	30/50	0	30/50	53/100	47/100	100/100	
Exp.3				80/100	8/100	88/100	
**Total**			**62/150 (41%)**			**288/300 (96%)**	**0.0001**
	**25°C for 2 months**						
Exp.1	2/100	0	2/100	2/100	0	2/100	
Exp.2	1/100	2/100	3/100	2/100	0	2/100	
Exp.3	2/100	0	2/100	4/100	0	4/100	
**Total**			**7/300 (2.3%)**			**8/300 (2.6%)**	
	**37°C for 1 month then 25°C for 1 month**						
Exp.1	2/100	2/100	4/100	33/100	34/100	67/100	
Exp.2	2/100	2/100	4/100	13/100	4/100	17/100	
Exp.3	3/100	4/100	7/100	16/100	1/100	17/100	
Total			**15/300 (5%)**			**101/300 (34%)**	**0.0001**

#### *Adults*

In the first batch of adults, which was maintained at 4°C for one month and then transferred at 25°C for rabbit attachment, 10–40% infected ticks died compared to a lack of death for non-infected ticks (30/120 vs. 0/80, p = 0.0001). No difference was observed between the infected and non-infected adults for the second batch, which was maintained at 25°C (2/120 vs. 0/120). In the third batch, which was maintained at 37°C for one month, 37.5–85% of infected ticks and 15–40% of non-infected ticks died (75/120 vs. 29/120, p = 0.0001). Among all of the dead ticks, 25–75% of infected ticks died before attachment to the rabbit, and 10–20% of non-infected ticks died before attachment to the rabbit (Table 2).

**Table 2:** The effect of temperature on *R. conorii conorii*-infected and non-infected *Rhipicephalus sanguineus* adults.

ADULTS							
	Non-infected			*R. conorii*-infected			p values (total deaths non-infected/infected)
	Deaths before attach	Deaths after attach	Total deaths (%)	Deaths before attach	Deaths after attach	Total deaths (%)	
	**1 month at 4°C then 25°C for 4 days**						
Exp.1	0	0	0/40	2/40	2/40	4/40	
Exp.2	0	0	0/40	10/40	0	10/40	
Exp.3				3/40	13/40	16/40	
**Total**			**0/80**			**30/120 (25%)**	**0.0001**
	**25°C for 1 month**						
Exp.1	0	0	0/40	0	0	0/40	
Exp.2	0	0	0/40	0	0	0/40	
Exp.3	0	0	0/40	0	2/40	2/40	
**Total**			**0/120**			**2/120 (1.6%)**	
	**37°C for 1 month**						
Exp.1	8/40	8/40	16/40	20/40	6/40	26/40	
Exp.2	4/40	2/40	6/40	10/40	5/40	15/40	
Exp.3	4/40	3/40	7/40	30/40	4/40	34/40	
**Total**			**29/120 (24%)**			**75/120 (62.5%)**	**0.0001**

In conclusion, infected ticks (nymphs and adults) had a comparatively higher rate of mortality at 37°C than at 25°C, and after a 4°C cooling when compared to non-infected ticks. Moreover, only engorged nymphs and adults survived at high and low temperatures.

## Discussion

This study confirms the vertical transmission of *R. conorii conorii* in naturally infected *Rh. sanguineus* ticks over twelve generations with a TOT rate of 100% and an FIR of up to 99%. *R. conorii conorii* was detected in ovary tissue by electron microscopy and by Gimenez staining, which supports the mechanism of transmission through several generations of infected ticks. The duration of the different steps of the tick life cycle in laboratory conditions were similar between non-infected and *R. conorii conorii*-infected ticks. These results are in agreement with recent published data about non-infected *Rh. sanguineus* [27]. The difference in the average weights of engorged females and eggs between the infected and non-infected ticks suggests that the fecundity of infected female ticks is lower than that of uninfected females. This implies that the prevalence of infection in a tick population should gradually decline and disappear without periodical augmentation.

The mortality rate of infected and non-infected engorged nymphs and adults maintained in our laboratory at 25°C and 80% RH was approximately 2%. In contrast, a comparatively higher mortality rate was observed when *R. conorii conorii*-infected engorged nymphs (88–10%, 4°C; 17–67%, 37°C) and adults (10–40%, 4°C; 37.5–85%, 37°C) that were exposed to low temperature or high temperature for one month were transferred to 25°C, compared to the control group (Table 1, 2). The negative effect of temperature on the viability of *Rh. sanguineus* infected with *Rickettsia conorii conorii* could be related to the long-recognized phenomenon known as reactivation, which remains poorly understood [28]. Between 1926 and 1930, Spencer and Parker demonstrated that triturated and starved *Dermacentor andersoni* ticks infected with *Rickettsia rickettsii*, the agent of Rocky Mountain spotted fever, did not cause disease but did result in seroconversion when injected into guinea pigs. However, feeding the ticks for a short time or keeping them at an elevated temperature (24 to 48 h at 37°C before trituration and inoculation) resulted in clinical manifestation of disease. These authors postulated that the virulence of *R. rickettsii* in the tick vector is linked directly to the physiological state of the tick and defined this phenomenon as “reactivation” [29]–[32]. In 1982, reversible structural modifications of *R. rickettsii* were demonstrated to be linked to physiological changes in the tick host and correlated with reactivation, i.e., the restoration of pathogenicity and virulence infectivity [28], [33]. More recently, *R. rickettsii* was shown to be lethal for the majority of experimentally and transovarially infected *D. andersoni* [18]. However, infected female ticks incubated at 4°C presented a lower mortality rate than those held at 21°C or 27°C. Although temperature is a common environmental signal for the upregulation of virulence gene expression, the information currently available in the literature poorly explains the reactivation phenomenon and its consequences for ticks [34].

In the present experiments, the temperature range (4°C, 25°C and 37°C) approximated the temperature differentials expected to be encountered by *Rh. sanguineus* in the natural environment in southern France and more generally, in Mediterranean settings. As confirmed in our study, infected and non-infected eggs, larval and nymphal unfed stages are unable to survive at a cold temperature in laboratory conditions, and the temperature exerts considerable influence on the length of their life cycle [27], [35]. Recently, the effect of low temperature (8±2°C) on non-infected *Rh. sanguineus* eggs has been shown to be a major limiting factor for the establishment of populations of the tick in colder regions [36]. Non-infected engorged nymphs and adults are less influenced by daily temperature. The maximum survival of nymphs and adult ticks occurs at 20–30°C and 85% relative humidity; the minimal temperature threshold for molting is between 10 and 15°C [17]. *Rh. sanguineus* overwinter as engorged nymphs or unfed adults [35], so our preliminary results suggest that infected ticks might not survive the winter. This could help to explain the scarcity of infected ticks found in the wild. Further studies investigating whether the mortality of *R. conorii conorii*-infected ticks is higher among diapaused ticks would be interest and could have important implications for the ecology of MSF. Such studies could be performed by exposing ticks to natural conditions or simulating natural conditions with proper regimens of photoperiod and temperature, as the diapause is induced where temperature is still warm, but changes in photoperiod induces ticks to enter a state of dormancy in a safe place, in order to survive to adverse conditions that will come the next winter.

In regards to the ecology of Rocky Mountain spotted fever, it is generally hypothesized that *R. rickettsii* is maintained in nature by the regular establishment of new populations of infected ticks. The probability of new populations of ticks becoming infected with *Rickettsiae* is difficult to precisely calculate, but a rough estimate can be obtained based on the assumed life span of susceptible mammals, the antibody prevalence in mammals, the average number of days of peak rickettsemia in infected animals and the number of days of infectious feeding on rickettsemic animals required to establish generalized infections in ticks [37]. It is likely that vertebrate reservoirs play a more dominant role in the ecology of *R. conorii conorii* than previously thought. Non-immune dogs, which include puppies in endemic areas or dogs living outside endemic areas of MSF or, at least, *Rh. sanguineus*, have been suggested as potential reservoirs for *R. conorii conorii* [1]. Recently, Levin et al. [38] reported that dogs are capable of acquiring *R. conorii israelensis* from experimentally infected *Rh. sanguineus* ticks that could also transmit infection to cohorts of uninfected ticks. Other animals have also been found to be experimentally susceptible to *R. conorii*, such as hedgehogs, Swiss mice, Hartley guinea pigs and *Spermophilus citellus* (*Citellus citellus*) [1]. Recently, one of 16 *Rh. sanguineus* collected from hedgehogs tested positive for *R. conorii* [39]. In addition, the role of the European rabbit *Oryctolagus cuniculus* in the epidemiology of MSF had been suggested by pioneering rickettsiologists [1]. Rabbit ticks and fleas, as well as that of small rodents such as *Pitymys duodecimcostatus* living in rabbits burrows, are suspected to be involved in the *R. conorii conorii* life cycle. Interestingly, the prevalence of infected *Rh. sanguineus* may vary from one specific setting to another within endemic areas, and the foci of MSF are usually small with a low propensity for diffusion [1]. However, a reservoir role has not been confirmed for any of these animals [1].

More work is needed to thoroughly decipher the relationship between *R. conorii conorii* and its vector, *Rh. sanguineus*. Aside from the need to definitively confirm the role of animal reservoirs in perpetuating *R. conorii conorii*, continued investigations of the *Rh. sanguineus*-*R. conorii conorii* interaction are needed to provide a better understanding of the factors influencing the ecology and epidemiology of MSF. In particular, studies on the poorly understood rickettsial inactivation-reactivation phenomenon may provide a better insight into the interaction between *Rickettsiae* and ticks.
